# A study of conjunctival impression cytology in patients undergoing allogeneic hematopoietic stem cell transplantation and its relationship with Ocular Graft versus Host Disease

**DOI:** 10.22336/rjo.2025.12

**Published:** 2025

**Authors:** Thanuja Gopal Pradeep, Deepthi Rameshbabu Honniganur, Santhosh Kumar Devadas

**Affiliations:** Department of Ophthalmology, MS Ramaiah Medical College Hospital, Bangalore, India

**Keywords:** Ocular Graft versus Host disease, allogeneic hematopoietic stem-cell transplantation, CIC = Conjunctival Impression Cytology, HSCT = Hematopoietic Stem Cell Transplantation, oGVHD = Ocular Graft Vs. Host Disease, allo-HSCT = Allogenic Hematopoietic Stem Cell Transplantation, OSDI = Ocular Surface Disease Index

## Abstract

**Purpose:**

To assess the proportion of patients with dry eye syndrome and to examine the changes in conjunctival impression cytology (CIC) in all patients undergoing hematopoietic stem cell transplantation (HSCT) by employing CIC as a diagnostic tool for ocular graft vs. host disease (oGVHD).

**Materials and methods:**

Every patient who received HSCT underwent a thorough ophthalmic examination, which included visual acuity, an assessment of dry eyes using objective tests such as Schirmer’s I test, tear film break-up time, and subjective tests such as the Ocular Surface Disease Index (OSDI) questionnaire. Conjunctival impression cytology was conducted after that, following informed consent.

**Results:**

This study included 24 eyes from 12 patients who underwent allogeneic hematopoietic stem cell transplantation (HSCT), with a mean age of 31.4 ± 11.06 years. Dry eye disease was observed in 28.8% of the cases. Based on the symptoms, 16 eyes (66.67%) were diagnosed with oGVHD. The Ocular Surface Disease Index (OSDI) indicated mild symptoms in 4 eyes (16.67%), moderate symptoms in 11 eyes (45.33%), and severe symptoms in 1 eye (4.17%) in individuals with ocular GVHD. In contrast, 93.55% of eyes without oGVHD exhibited mild symptoms, while 6.71% showed moderate symptoms (p = 0.002).

Objective assessments indicated that Schirmer’s I score was ≤ 5 mm in 50% of the eyes (n = 12), and tear film breakup time was less than 5 seconds (3.85 ± 2.18 seconds) in 29.17% of eyes with oGVHD (p = 0.05). The conjunctival impression cytology (CIC) was abnormal in 9 eyes (37.5%, p = 0.05), revealing changes in cell morphology, such as decreased goblet cell density, reduced cytoplasmic mucin, and inflammatory cells. The average goblet cell density was measured at 190.63 ± 81.00 cells/mm^2^ in 6 eyes (p = 0.05), showing a correlation with the time since HSCT; specifically, when the interval from HSCT to CIC assessment was 40.67 ± 5.01 months, the goblet cell density significantly decreased to 181.00 ± 76.62 (p = 0.04).

Changes in morphology were observed in 8 eyes with oGVHD (91.7%) compared to 2 eyes without oGVHD (16.67%), with abnormal CIC results in 66.67% of cases (p = 0.02).

**Discussion:**

This study highlights the significant prevalence of dry eye disease (DED) and ocular surface alterations in patients undergoing allogeneic hematopoietic stem cell transplantation (allo-HSCT), particularly in those diagnosed with ocular graft-versus-host disease (oGVHD). The findings align with previous research, indicating that oGVHD is a leading cause of post-HSCT ocular morbidity, with symptoms ranging from mild irritation to severe ocular surface damage. The significantly higher Ocular Surface Disease Index (OSDI) scores, reduced Schirmer’s I test values, and shorter tear film breakup times (TBUT) in oGVHD patients corroborate established diagnostic criteria for severe tear dysfunction. Moreover, conjunctival impression cytology (CIC) revealed marked goblet cell loss and squamous metaplasia, consistent with prior studies linking these changes to chronic inflammation and epithelial instability in oGVHD. The correlation between longer post-HSCT duration and further reduction in goblet cell density underscores the progressive nature of ocular surface damage in these patients. Given its non-invasive nature and diagnostic accuracy, CIC is a valuable tool for early oGVHD detection, facilitating timely therapeutic intervention and potentially mitigating long-term ocular complications.

**Conclusion:**

Significant alterations in the ocular surface were noted in those who had chronic oGVHD and had undergone allo-HSCT. The Ocular Surface Disease Index (OSDI) score, corneal involvement severity, and Schirmer I test results are key indicators of ocular involvement following allogeneic hematopoietic stem cell transplantation (allo-HSCT). The most notable abnormalities observed in the conjunctival impression cytology of eyes with oGVHD include squamous cell metaplasia and reduced goblet cell density, with lesser changes noted in eyes without oGVHD. Therefore, conjunctival impression cytology is advisable for patients who have received allogeneic HSCT to facilitate the early diagnosis of oGVHD.

## Introduction

Graft-versus-host disease (GVHD) is a frequent complication of allogeneic transplants. It involves a set of clinical symptoms that arise when donor lymphocytes attack host antigens, a reaction that typically occurs after allogeneic stem cell transplantation. Chronic GvHD mainly presents as “sicca syndrome”, wherein patients present with dryness of mucous membranes. The occurrence of GvHD not only contributes enormously to transplantation-related morbidity and mortality but also has several psychological implications for the patient [1].

Allogeneic hematopoietic stem cell transplants, such as bone marrow, peripheral blood stem cell, and cord blood cell transplants, represent the only curative options for numerous hematological disorders. Despite being effective modalities, these transplants nevertheless contribute significantly to patient morbidity. The donor’s immune system perceives the recipient’s tissue as alien, contributing to the pathophysiology of GVHD. Inflammation brought on by GVHD may lead to fibrosis. According to published reports, 40% of allo-HSCT patients experience ocular (oGVHD), which lowers their quality of life [2-4].

Patients undergoing allo-HSCT and those receiving lymphocyte infusions may develop acute GVHD/chronic GCHD or an overlap syndrome, which has features of both acute and chronic GVHD [5].

Ocular complications can develop following hematopoietic stem cell transplantation (HSCT), and dry eye constitutes very distinctive signs and symptoms for the diagnosis of chronic GVHD (cGVHD) [6]. Ocular GvHD predominately presents as dry eyes due to ocular surface changes. The inflammation can eventually lead to a decreased density of goblet cells and can also cause scarring, especially of the lacrimal glands, conjunctiva, and cornea [7,8].

In this study, we analyzed the ocular surface changes in patients who underwent HSCT and estimated the proportion of dry eye occurrence in such individuals. Conjunctival impression cytology was performed in all patients to look for ocular surface changes.

## Materials and methods

The study included 24 eyes of 12 patients undergoing allogeneic HSCT at the medical oncology department between November 2020 and March 2022. An institutional Ethics Committee approval was obtained. Before the tests, informed consent was obtained from each patient.

All patients who underwent allogeneic HSCT (>3 months) were included in the study, and those on chronic ocular medications, who had any ocular surgery in the last 3 months, and any prior ocular surface disorders, like exposure keratitis, were excluded.

The participants had a thorough ophthalmic examination, including visual acuity, the Schirmer’s I test for dry eyes (without instilling anaesthetic drops), and Tear Film Break-up Time (TBUT).

Patients completed the Ocular Surface Disease Index (OSDI) questionnaire to assess better subjective symptoms, a standard 12-item tool used to determine the OSDI score. The scores were categorized as normal (0-12), mild (13-22), moderate (23-32), or severe (33-100), depending on the total points obtained [9]. The questionnaire was based on three subscales: vision-related function, ocular symptoms, and environmental triggers. According to the Dry Eye Workshop Recommendations (DEWS), the diagnosis of dry eye was based on TBUT values (<5 seconds) and Schirmer’s I test results (<5 mm) after 5 minutes [10].

Conjunctival Impression Cytology (CIC) was obtained from the bulbar conjunctiva’s nasal region. The conjunctival cytology sample was collected using a surfactant-free Millipore cellulose acetate filter paper with a pore size of 0.45 μm (47 mm diameter). Using a sterile blade, the created circular discs were fashioned into 5 x 50 mm rectangular strips. After administering a single drop of local anaesthetic, the eye’s surplus tear film and medicine were removed. The bulbar conjunctiva’s nasal portion received the filter paper’s application. The filter paper was gently pressed onto the ocular surface using a sterile cotton bud. After roughly 5 to 10 seconds, the paper was left in contact with the eye and removed using forceps. The paper was quickly moved to a glass slide and fixed for ten minutes using a fixative solution containing formaldehyde, ethyl alcohol, and glacial acetic acid in a 1:1:20 volume ratio. Hematoxylin and PAS stains were employed. Conjunctival cytology was evaluated for goblet cell density and was assessed by a single expert pathologist, and grading was done according to the Nelson and Wright classification [2]. Any grade higher than two was considered as aberrant.

## Results

### Demographic data

A total of 12 patients post-allogenic-HSCT were included, with a mean age of 31.4 ± 11.06 years; 8 males (mean age 31.2 ± 10.65) and four females (mean age 30.2 ± 11.78) were evaluated. All 10 had undergone bone marrow transplantation (BMT) - a human leucocyte antigen-matched, sibling allogeneic transplant. The mean time of recruitment of the patients into the study post-allo-HSCT was 40.67 ± 5.01 months. The indications for allo-HSCT are shown in **Table 1**.

**Table 1:** Indications for allo-HSCT

Sl. number	Diagnosis	Number (percentage)
1	Aplastic anaemia	4 (33.33%)
2	Chronic Myeloid Leukaemia	3 (25%)
3	Acute Myeloid Leukaemia	2 (16.67%)
4	Acute Lymphoblastic Leukaemia	1 (8.33%)
5	Myelodysplastic Syndrome	1 (8.33%)
6	Pure red cell aplasia	1 (8.33%)

### Ocular Surface Evaluation tests data

Chronic oGVHD was observed in 66.67% (n = 16) of allo-HSCT patients.

The OSDI score was mild in 16.67% (n=4), moderate in 45.83% (n=11), and severe in 4.17% (n=1) of those with ocular GVHD (oGVHD) eyes. Similarly, it was found to be mild in 93.55% and moderate in 6.71% of those without ocular-GVHD eyes (p = 0.002).

Schirmer’s I test score: The mean Schirmer I test score (without anaesthetic instillation at the end of 5 minutes) was 18.67 ± 10.85 mm (range 0-35 mm), of which 50% (n = 12) had a Schirmer I test of <5 mm. 6 eyes had Schirmer’s value of 22.35 ± 1.49, 4 eyes had Schirmer’s of 12.00 ± 1.39 (p = 0.06), and 14 eyes had 6.36 ± 2.70 (p = 0.05). Fluorescein tear break-up time (TBUT): Tear film breakup time in these patients was found to be less than 5 seconds (3.85 ± 2.18 seconds) in 33.33% (n = 4) (p = 0.05).

The above values are shown in the table below (**Table 2**).

**Table 2:** OSDI scores, Schirmer test scores, and TBUT values and their correlation with the severity of dry eye

Grading	Number of eyes	OSDI score	Schirmer’s test (in mm at 5 mins.)	TBUT (in secs.)
Normal	6	10.34 ± 1.26	22.35 ± 1.49	14.92 ± 1.17
Mild	4	20.67 ± 1.34	12.00 ± 1.39 *(p=0.06)*	9.23 ± 1.22
Moderate-Severe	14	64.22 ± 10.58	6.36 ± 2.70 *(p = 0.05)*	3.85 ± 2.18 *(p=0.05)*

### Conjunctival Impression Cytology

8 CIC smears (n = 24) out of the 24 total CIC smears analyzed presented cytology changes: 4 showed grade 1 changes, 11 showed grade 2 changes, and 1 showed grade 3 changes (grades are presented in **Table 3**, as determined by Nelson’s scoring system). Anything above or comparable to grade 2 was deemed aberrant.

**Table 3:** Nelson’s scoring system

GRADE	FEATURES
0	>500 cells/mm2
1	500-300 cells/mm2
2	300-100 cells/mm2
3	<100 cells/mm2

Dry eye disease associated with chronic ocular GVHD was observed in 16 eyes (66.67%), with 15 eyes (62.5%) presenting moderate to severe dry eye and four eyes (16.7%) presenting mild dry eye. Based on the ocular surface evaluation test results, the eyes were classified as oGVHD and non-oGVHD. The ocular surface evaluation data between eyes with oGVHD and eyes without oGVHD were analyzed.

**Fig. 1:**
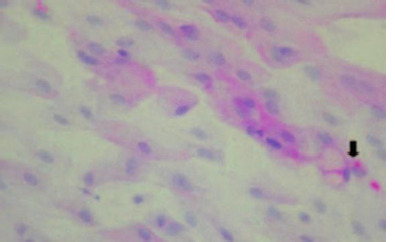
CIC from a 39-year-old male with GVHD-related severe dry eye (AML) showing decreased GCD and increased inflammatory cells. The black arrow shows goblet cells with reduced cytoplasmic mucin

Overall statistical analysis of the association of CIC of eyes of post-allo-HSCT patients with the various ocular surface evaluation parameters showed a statistically significant association of CIC with Schirmer I test score (p = 0.05) and OSDI (p = 0.04) presented in **Table 2**. The CIC was abnormal in 66.67% with altered morphology noted in 8 eyes with oGVHD (91.7%) and two eyes without oGVHD (16.67%) (p = 0.02). A comparison of CIC changes and dry eyes was also done, as shown in **Table 4. Fig. 1** shows decreased goblet cell density (GCD) in a 39-year-old male patient diagnosed with Acute Myeloid Leukemia (AML) (Sl. no. 6).

**Table 4:** Comparison between dry eye severity and CIC changes

Characteristics	Normal	Mild dry eye	Moderate-severe dry eyes
GCD (cells/mm^2^)	1212.13 ± 31.82	701.30 ± 63.11 *(p=0.05)*	390.63 ± 81.00 *(p=0.05)*
Squamous cell metaplasia (Nelson’s score)	0.56 ± 0.32	0.59 ± 0.56 *(p=0.22)*	0.63 ± 0.74 *(p=0.06)*

## Discussion

The extent of involvement of the ocular surface tissues, including the tear film, cornea, conjunctiva, lids, and lacrimal glands, determines the severity of ocular surface disease in chronic ocular GVHD. Patients with ocular cGVHD have conjunctiva that exhibits T-cell-related immune-inflammatory processes, apoptosis, fibrosis, and TH1-associated chemokines, all of which contribute to ocular surface morbidity in chronic GVHD [2,6]. This leads to several consequences, such as inflammation and cicatrization of the conjunctiva, disruption of the mucus layer in the tear film due to decreased goblet cell density, lacrimal gland dysfunction caused by inflammation and fibrosis of the lacrimal gland, which changes the integrity of the aqueous layer in the tear film, and instability of the lipid layer in the tear film due to meibomian gland dysfunction [3]. Patients with cGVHD have been shown to have altered conjunctival mucosal microvilli in both number and form.

Despite this, there was no discernible variation in the cumulative incidence of cGVHD among recipients of post-allogeneic hematopoietic stem cell therapy. Dry eye is the most prevalent clinical finding in roughly 5-34% of individuals [11].

Reports on ocular surface illness after allogeneic-HSCT with nonmyeloablative conditioning regimens are sparse. In a prospective study [2] of 101 hematologic patients following allogeneic-HSCT, the development of oGVHD was observed in 54% of the patients. Five allo-HSCT recipients in our study developed persistent systemic GVHD. A mean of 24.67 ± 2.83 months had passed since allo-HSCT for GVHD to manifest, and eight eyes (33.33%) developed chronic oGVHD. In our analysis, individuals undergoing allogeneic hematopoietic stem cell transplantation who had chronic oGVHD accounted for 45.56% of cases of chronic systemic GVHD. This supports previous research [1,1], which has shown a robust correlation between chronic systemic GVHD and chronic oGVHD. Ocular involvement in GVHD in allo-HSCT patients has also been extensively studied [6]. The prevalence of dry eye illness in patients with GVHD, along with dry mouth and a Schirmer test value of oGVHD, has been linked to several risk factors, including an older age group (>27 years), peripheral blood stem cell transplant, chronic GVHD, and chronic/acute myeloid leukaemia [2,1,2].

In our study, the CIC had aberrant morphology in 76.56% of eyes with oGVHD and 14.58% without oGVHD. The continued subclinical immune injury could explain this by targeting tissues on the ocular surface in the eyes of post-allogeneic HSC individuals who have not experienced oGVHD.

Since no other group was in our investigation, we could not compare our results to the prevalence of CIC alterations in healthy eyes. Understanding the relationships between CIC changes in oGVHD eyes could have been aided by evaluating fluorescein staining (FS), measuring tear meniscus height (TMH), CIC in other quadrants of the bulbar conjunctiva, tear osmolarity, and corneal sensations in addition to the baseline characteristics of dry eye disease.

The presence of oGVHD was linked to several previously described characteristics, including FS, TMH, and CIC, in distinct quadrants of the bulbar conjunctiva (except from the nasal region). These findings were included in research conducted by Murugesan et al. [1,3]. An additional investigation on the issue by Wang et al. provided baseline profiles of the ocular surface and tear dynamics of oGVHD and non-oGVHD-related dry eye sickness in allo-HSCT patients [6]. To investigate meibomian gland obstruction, tear evaporation rate, corneal sensitivity, Schirmer’s I test, TBUT, ocular surface vital staining, CIC, and brush cytology, they examined 50 eyes from 25 post-allo-HSCT patients and 28 controls. They reported altered conjunctival epithelial shape, decreased conjunctival goblet cell density, and elevated inflammation in chronic GVHD-related dry eyes.

In addition, they observed extensive changes to the ocular surface in individuals who had undergone allo-HSCT, regardless of whether they had chronic dry eye caused by GVHD. Our research also reached the same conclusion: the course of chronic GVHD-related dry eye is significantly influenced by the degree of the inflammatory process. To further understand the immunopathogenic mechanisms causing ocular surface morbidity in chronic GVHD, a new study is still looking for efficient therapy and prevention strategies [12]. For patients with oGVHD, the Schirmer I test and the OSDI scores are valuable indicators of dry eye illness. In patients with persistent GVHD who have had allo-HSCT, ocular surface changes are observed in 30% of the examined cases. With changed morphology observed in 8 eyes with oGVHD (91.7%) and two eyes without oGVHD (16.67%), the CIC was abnormal in 66.67% of cases (p = 0.02).

Additional research on other advanced dry eye disease criteria may help us comprehend the immunopathogenesis behind GVHD-related dry eye disease.

## Conclusion

Patients who have received allogeneic hematopoietic stem cell transplantation (allo-HSCT) may develop chronic ocular graft-versus-host disease (oGVHD), which is characterized by profound changes to the ocular surface. In eyes after allogeneic-HSCT, the OSDI score, the degree of corneal involvement, and the results of the Schirmer I test function as indicators of ocular involvement. Furthermore, conjunctival impression cytology abnormalities, including squamous cell metaplasia and decreased goblet cell density, were observed in eyes with oGVHD. Conjunctival impression cytology is, therefore, wise in the early diagnosis of oGVHD in individuals with allogeneic HSCT.
